# WE ARE NOT CURING A LOT OF THINGS, BUT WE ARE CARING: GERIPACT PATIENT NEEDS SHAPE TEAM NEEDS

**DOI:** 10.1093/geroni/igz038.2774

**Published:** 2019-11-08

**Authors:** Samantha Solimeo, Melissa J Steffen, Ellen E Gardner, Omonyele Adjognon, Marlena Shin, Jennifer Moye, Jennifer L Sullivan

**Affiliations:** 1 Center for Access and Delivery Research and Evaluation Primary Care Analytics Team Iowa City VA Health Care System, Iowa City, Iowa, United States; 2 Center for Access & Delivery Research & Evaluation (CADRE) Primary Care Analytics Team, Iowa City, Iowa, United States; 3 Carver College of Medicine, Iowa City, Iowa, United States; 4 Center for Healthcare Organization & Implementation Research, Boston, Massachusetts, United States; 5 New England Geriatric Research, Education, and Clinical Center Department of Psychiatry; Harvard Medical School, Boston, Massachusetts, United States; 6 Center for Healthcare Organization and Implementation Research, Boston University School of Public Health, Boston, Massachusetts, United States

## Abstract

This study used a qualitative observational design to identify the team-, clinic-, and system-level resources necessary for effective geriatric medical home (i.e., GeriPACT) implementation and to differentiate the needs of GeriPACT compared to traditional PACT. Analysis of 80 interviews conducted with team members from 8 geographically dispersed GeriPACTs identified needs that may be unrecognized by primary care leadership, including: clinical space to accommodate caregivers and patients with impaired visual, mobility, cognitive, or hearing acuity; greater utilization of caregiver support programs and social workers to facilitate aging-in-place; age-sensitive clinical reminders; team member continuity and direct phone lines to reduce patient anxiety; and longer standard appointment lengths to reflect clinical complexity. In contrast to traditional primary care teams, GeriPACTs are not simply “PACTs for older adults”: GeriPACT members articulate population-specific resources that require support from facility leadership to accommodate the complex, age- clinical and social resources needed to support aging-in-place.

